# Analysis of Global Collection of Group A *Streptococcus* Genomes Reveals that the Majority Encode a Trio of M and M-Like Proteins

**DOI:** 10.1128/mSphere.00806-19

**Published:** 2020-01-08

**Authors:** Hannah R. Frost, Mark R. Davies, Valérie Delforge, Dalila Lakhloufi, Martina Sanderson-Smith, Velusamy Srinivasan, Andrew C. Steer, Mark J. Walker, Bernard Beall, Anne Botteaux, Pierre R. Smeesters

**Affiliations:** a Molecular Bacteriology Laboratory, Université Libre de Bruxelles, Brussels, Belgium; b Department of Microbiology and Immunology, University of Melbourne at the Peter Doherty Institute for Infection and Immunity, Melbourne, Australia; c Illawarra Health and Medical Research Institute and Molecular Horizons, School of Chemistry and Molecular Bioscience, University of Wollongong, Wollongong, Australia; d National Center for Immunization and Respiratory Diseases, Centers for Disease Control and Prevention, Atlanta, Georgia, USA; e Tropical Diseases Research Group, Murdoch Children’s Research Institute, Melbourne, Australia; f Australian Infectious Diseases Research Centre and School of Chemistry and Molecular Biosciences, University of Queensland, St Lucia, Australia; g Academic Children Hospital Queen Fabiola, Université Libre de Bruxelles, Brussels, Belgium; h Centre for International Child Health, University of Melbourne, Melbourne, Australia; University of Wisconsin—Madison

**Keywords:** Streptococcus pyogenes, Mga regulon, M-like proteins, global GAS diversity

## Abstract

The core Mga (multiple gene activator) regulon of group A *Streptococcus* (GAS) contains genes encoding proteins involved in adhesion and immune evasion. While all GAS genomes contain genes for Mga and C5a peptidase, the intervening genes encoding M and M-like proteins vary between strains. The genetic make-up of the Mga regulon of GAS was characterized by utilizing a collection of 1,688 GAS genomes that are representative of the global GAS population. Sequence variations were examined with multiple alignments, and the expression of all core Mga regulon genes was examined by quantitative reverse transcription-PCR in a representative strain collection. In 85.2% of the sampled genomes, the Mga locus contained genes encoding Mga, Mrp, M, Enn, and C5a peptidase proteins. These isolates account for 53% of global infections. Only 9.1% of genomes did not contain either an *mrp* or an *enn* gene. The pairwise identity within Enn (68.6%) and Mrp (83.2%) protein sequences was higher than within M proteins (44.7%). Gene expression varied between strains tested, but high expression was recorded for all genes in at least one strain. Previous nomenclature issues were clarified with molecular gene definitions. Our findings support a shift in focus in the GAS research field to further consider the role of Mrp and Enn in virulence and vaccine development.

**IMPORTANCE** While the GAS M protein has been the leading vaccine target for decades, the bacteria encode many other virulence factors of interest for vaccine development. In this work, we show that *emm*-like genes are encoded in a remarkable majority of GAS genomes and expressed at a level similar to that for the *emm* gene. In collaboration with the U.S. Centers for Disease Control, we developed molecular definitions of the different *emm* and *emm*-like gene families. This clarification should abrogate mistyping of strains, especially in the area of whole-genome typing. We have also updated the *emm*-typing collection by removing *emm*-like gene sequences and provided in-depth analysis of Mrp and Enn protein sequence structure and diversity.

## Abstract (precis)

While the GAS M protein has been the leading vaccine target for decades, the bacteria encode many other virulence factors of interest for vaccine development. In this work, we show that *emm*-like genes are encoded in a remarkable majority of GAS genomes and expressed at a level similar to that for the *emm* gene. In collaboration with the U.S. Centers for Disease Control, we developed molecular definitions of the different *emm* and *emm*-like gene families. This clarification should abrogate mistyping of strains, especially in the area of whole-genome typing. We have also updated the *emm*-typing collection by removing *emm*-like gene sequences and provided in-depth analysis of Mrp and Enn protein sequence structure and diversity.

## INTRODUCTION

Group A *Streptococcus* (GAS) is a human-specific bacterial pathogen responsible for a range of different conditions affecting different tissue types, including pharyngeal epithelium, keratinocytes, and deeper tissues during invasive diseases (1). GAS infection also leads to autoimmune sequelae, such as rheumatic heart disease, which is responsible for around 320,000 of the more than 500,000 deaths worldwide each year attributed to GAS (2).

GAS produce many virulence factors to aid in establishing and propagating human infection. The GAS M protein is an important and well-characterized virulence factor that is crucial for adhesion to primary tissue sites, invasion of nonimmune cells, and evasion of the host immune system (3). The gene encoding M protein, *emm*, is found in the Mga (multiple gene activator) regulon. Each GAS strain carries a single variant of the *emm* gene, whose sequence of the 5′ 180 nucleotides forms the basis for the *emm*-typing scheme (4, 5). *emm* typing has differentiated more than 220 *emm* types to date (6). A convenient feature of *emm* genes is that their signal sequence encoding regions universally contain a conserved 19-bp sequence (7). This feature has greatly simplified the assignment of *emm* types from short-read genomic sequences since they are invariably very closely linked to the upstream 19-bp primer 1 sequence (8). Genomic sequencing has also revealed that certain historically established *emm* types have been assigned to sequences located in *emm*-like genes due to the annealing of *emm*-typing primers to *emm*-like genes (9). A more recent classification, called the *emm*-cluster-typing scheme, groups strains into 48 clusters based on full M protein sequence homology and functional properties (10–12).

The core Mga regulon spans from the ubiquitous genes for Mga (*mga*), a transcriptional regulator, to the C5a peptidase (*scpA*) (13). Other genes that can be present in the Mga regulon include genes encoding the M-like proteins (*emm*-like genes), Mrp (*mrp*), and Enn (*enn*). In some genomes the locus also encodes protein H (*sph*), a surface protein involved in immune evasion (14); streptococcal inhibitor of complement (SIC; *sic*), a secreted virulence factor also involved in immune evasion (15); and proteins closely related to SIC (CRS; *crs*) and distantly related to SIC (DRS; *drs*) (16). Depending on the *emm*-like gene content of this locus, strains are classified into five *emm* patterns (A to E) (17).

As well as regulating Mga regulon gene expression, the Mga protein directly affects transcription of genes involved in the early stages of infection and is chiefly active during the exponential growth phase. Mga indirectly affects expression of over 10% of the GAS genome (18, 19). Limited evidence suggests that *mrp* and *enn* genes are expressed between 4- and 32-fold less than the *emm* gene in the strains analyzed (20, 21).

M-like proteins are fibrillar coiled-coil proteins that extend from the surface of the bacteria and share structural characteristics similar to M proteins (22). The virulence potential of these proteins is relatively unclear, although they have been shown to share binding properties with M proteins (22). The vaccine potential of Mrp has been recently investigated, since antibodies against Mrp have been shown to elicit protection in animal models of infection (23) and increased the bactericidal activity of anti-M antisera (24).

In this study, we carefully describe the Mga core regulon of GAS based on a genetically diverse worldwide study of 2,083 GAS genomes (25). We applied particular emphasis to the genetic description of Mrp and Enn, since these two proteins remain poorly characterized to date. We also sought to address the mislabeling of certain *emm* types by molecular clarification of gene families.

(This information was presented in part at the 19th Lancefield International Symposium for Streptococci and Streptococcal Diseases in Fiji in 2017 and at the workshop of f-TALES: Big Data in Life Sciences and Biomedicine in Belgium in 2017.)

## RESULTS

**FIG 1:**
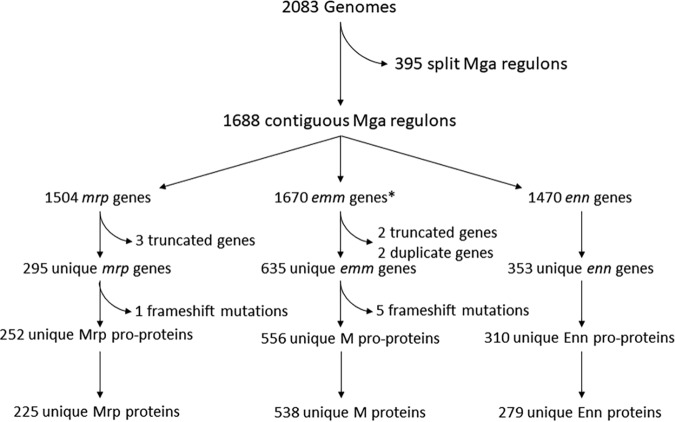
Details of the genome and gene collections. Details on the process by which the genomes and genes available were selected for further analysis. The final collection of alleles provides the best representation of global diversity of the gene families available, while avoiding the possibility of confounding by sequence ambiguities. *, a total of 19 genomes had substantial sequence ambiguities in the *emm* gene domain but nevertheless contained *emm*-typing sequences. These genes were excluded from gene family analyses, but genomes were included in Mga composition analyses.

The Mga regulon was located in a single contig in 1,688 genomes belonging to 130 different *emm* types, 39 *emm* clusters, and 262 phylogroups from the 2,083 global genome database (Fig. 1) (25).

### Defining *mrp*, *enn*, and *emm*.

*mga-*, *mrp-*, *emm-*, *enn-*, and *scpA*-specific oligonucleotide probes were designed to facilitate identification of gene families (Table 1). The *mrp* gene was defined as being an open reading frame (ORF) downstream of the *mga* gene, upstream of the *emm* gene, and containing the *mrp*-specific probe. The *enn* gene was defined as being an ORF downstream of the *emm* gene, upstream of the *scpA* gene, and containing the *enn* probe sequence. The *emm* gene was defined as containing either the *emm* probe 1 at the 5′ end or the *emm* probe 2 at the 3′ end of the gene. Chimeric *emm* genes were defined as containing the *emm* probe 1 at the 5′ end and the *enn* probe at the 3′ end of the gene. Sph genes were observed as large ORFs downstream of *emm* genes that did not contain either the *emm* or the *enn* probe and were identified by BLAST search.

**TABLE 1:** Nucleotide probes for *in silico* identification of Mga regulon genes

Gene	Flexibility^*a*^ (no. of nt)	Sequence	Probe in unique alleles (%)
*mga*	3	GAGATTGAAAAACAGTACGATGTTATCGTGACAGATGTTATGGT	386/390 (99.0)
*mrp*	1	AACCAAGAAAAAGAAAAGTTAGAAGC	295/295 (100)
*emm* (3*′*)	1	AACAAAGAGCTTGAAGAA	623/635 (98.1)
*emm* (5′)	0	TATTSGCTTAGAAAATTAA	624/635 (98.3)^*b*^
*enn*	1	TCTGAGTTAACRCAAGCRAARRYTCAACTYKY	350/352 (99.4)
*scpA*	1	GAAGTAACAGTAACAGTTCACAACAAATCTGATAAACCTCAAGAGTTGTATTA	550/553 (99.5)

a The flexibility number refers to the number of mismatched nucleotide (nt) bases allowed to provide 99 to 100% specificity and sensitivity. All *emm* genes contained at least one of the two *emm* probes; there were 32 genomes with 10 distinct *emm* alleles that contained the 3′ probe but not the 5′ probe and 18 genomes with 7 distinct *emm* alleles that contained the 5′ probe but not the 3′ probe.

b Whatmore et al. (7).

In the 1,688 genomes analyzed, there were 176 *emm* subtypes represented in more than one genome. Of these, more than one unique *mrp* allele was present in 46% of cases (*n* = 81) and more than one unique *enn* allele was present in 57% of cases (*n* = 100). Therefore, the previous nomenclature system in which the *emm*-like genes were named based on the *emm* type they were isolated from (26, 27) was not optimal. Thus, a systematic Mrp and Enn nomenclature was established, in which each unique protein sequence has a numerical identifier (e.g., Mrp1), and any allele that produces the same protein sequence is named as a subtype (e.g., Mrp1.1). This nomenclature is hosted on the website of the GAS reference laboratory at U.S. Centers for Disease Control and Prevention (CDC; https://www2a.cdc.gov/ncidod/biotech/strepblast.asp), and the alleles in each genome are listed in Table S3 in the supplemental material. Where possible, the association between previously designated *mrp* and *enn* sequences are noted in the lists of unique alleles (Table S4). However, since the new nomenclature is unlinked from the *emm* type of the strain, the previous names could unfortunately not be retained.

### Composition of Mga regulons.

Of the 1,688 genomes analyzed, all contain *mga* and *scpA* genes, which ranged from 6,016 to 11,641 bp apart. The length of each gene family within the core Mga regulon displayed some variability (Fig. S1), the most variable being *emm* and the genes encoding transposases, while the least variably sized genes were *mga* genes and *pgs* (X92371.1), a gene encoding Pgs, a 15.5-kDa protein of unknown function (CAA63115).

10.1128/mSphere.00806-19.1 FIG S1 Length and distribution of Mga regulon genes. Scatterplot comparing the range of sizes of gene families present in the Mga regulon. The central line represents the mean size, and the bars represent the SD and the colored circles indicate the range of sizes in the collection. Sizes ranged from the large *scpA* gene family (mean = 3,500 bp; SD = 92.9bp) to the small *pgs* gene family (mean = 461 bp; SD = 27.1 bp). The *emm* genes and *transposase* genes showed the most variability in length (*emm*: mean = 1,218 bp; SD = 153.6 bp; *transposase*: mean = 853 bp; SD = 256.5 bp); however, the latter is attributable to the presence of three major size variants. Download FIG S1, PDF file, 0.3 MB. Copyright © 2020 Frost et al. 2020 Frost et al. This content is distributed under the terms of the Creative Commons Attribution 4.0 International license.

**FIG 2:**
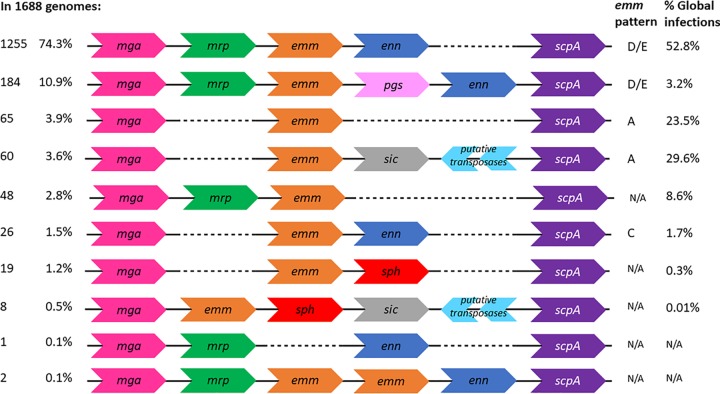
Configurations of Mga regulons. In the large collection of contiguous Mga regulon sequences, we identified 10 possible configurations of the regulon based on presence and positions of genes. All Mga regulons began and ended with the *mga* and *scpA* genes and could contain genes for Mrp, Emm, Enn, Pgs, protein H, SIC, and transposases. The most frequent Mga regulon configuration, with genes for the trio of M and M-like proteins, was present in around 74% of genomes, from *emm* types that are responsible for around half of global infections.

All of the genomes in the database possessed either an *emm* or *emm*-like gene, and the vast majority (85.2%) contained a gene for all three. Importantly, 74.3% of the genomes (*n* = 1,255) have a core Mga regulon consisting of: *mga*, *mrp*, *emm*, *enn*, and *scpA*, specifically in this stated order (Fig. 2). In the remaining 10.9% of isolates (*n* = 184), the Mga regulon also included a *pgs* gene between the *emm* and *enn* genes.

The 85.2% of genomes which contained genes for *mrp*, *emm* and *enn* would be a D or E pattern under the *emm*-pattern typing system. Pattern C, defined as containing an *emm* and *enn* gene, was present in only 1.5% of isolates (*n* = 25). Among the genomes without a gene encoding M-like proteins, around half (3.9% of total, *n* = 65) had only an *emm* gene between *mga* and *scpA* (*emm*-pattern A), while 3.6% (*n* = 60) also harbored genes for SIC and for two transposases downstream of the *emm* gene. The genomes containing *emm* but neither *enn* nor *mrp* genes belonged to only 15 different *emm* types, mostly belonging to A to C *emm* clusters (Table S5). There were no pattern B isolates within the collection, i.e., encoding two *emm* genes with no other M-like genes. A total of 47 genomes contained an *mrp* and *emm* gene but no *enn* gene. In two genomes there were two copies of the same *emm* gene between *mrp* and *enn* genes, and six genomes did not contain an *emm* gene but contained an *mrp* and *enn* gene. These variants have not previously been assigned to an *emm* pattern. Isolates with the same *emm* type tended to encode the same pattern of proteins in their Mga regulon.

A gene encoding protein H (*sph*) was present in 1.6% of isolates (*n* = 28), of which eight also contained a gene for SIC and genes for two transposases downstream. In all isolates containing a *sic* gene there were also genes present for two transposases (*n* = 67). The Mga regulons encoding protein H, transposases, SIC and *pgs* belonged to few *emm* types (Table S5).

Although Mga proteins are highly conserved (93.9% pairwise identity), we observed two distinct variants, which differ by 21% at the amino acid level (0.254 substitutions per site, Fig. S2A). The minor variant was present in 10.6% of genomes, exclusively in strains that do not encode an Mrp protein, i.e., strains from A and C patterns and strains encoding SIC and protein H. The other variant was present in the remaining 89.4% of genomes. There was very little diversity within each protein variant (98% sequence identity, 0.01 substitutions per site). The amino acid diversity of Mga proteins was more evenly distributed along the length of the proteins than the other proteins present in the Mga regulon (Fig. S2B). This is in concordance with previous findings of 24.5% nucleotide diversity between two divergent *mga* alleles (13, 28).

10.1128/mSphere.00806-19.2 FIG S2 Sequence identity within Mga regulon protein families. (A) Mga proteins are highly conserved (93.9% pairwise identity); however, two clear variants can be observed, MgaA and MgaB. MEGA was used to calculate genetic distances between all Mga proteins. Within both subgroups, the substitutions per site were 0.01 but between the groups was 0.25. (B) Details of the amino acid sequence identity within the different protein families present in the Mga locus. The percent identity is displayed along the length of mature protein consensus sequences. Mrp, M, Enn, and H proteins contain regions of low sequence identity in their N-terminal domains and become more conserved towards the C-terminal domains. Of the M and M-like proteins, Mrp proteins have the highest average pairwise identity (83.2%), followed by Enn proteins (68.6%), and M proteins have the lowest (44.7%). *pgs* sequences do not contain neither signal peptide (no YSIRK sequence) nor LPXTG motif indicative of streptococcal surface proteins. It is therefore unlikely to be a surface expressed virulence factor like many other Mga regulon-encoded proteins. Download FIG S2, PDF file, 1.4 MB. Copyright © 2020 Frost et al. 2020 Frost et al. This content is distributed under the terms of the Creative Commons Attribution 4.0 International license.

### *emm*.

**FIG 3:**
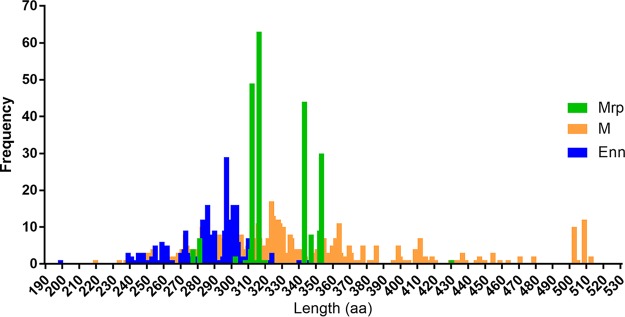
Distribution of M and M-like protein lengths. Distribution of the lengths of all M and M-like proteins from the collection. Bars represent the number of genomes in the collection that contained a protein of the size indicated on the *x* axis. M proteins show the most diversity in protein lengths and can be both the smallest or the largest of the trio of proteins. Enn proteins are typically smaller and have less variable length distribution, and Mrp proteins are largely restricted to four possible lengths.

The M protein has been genetically analyzed previously and was not a major focus of this study (6). The mature M protein sequences ranged in size from 220 to 513 amino acids in length (Fig. 3) and had an average pairwise identity of 44.7%.

### *mrp*.

An *mrp* gene was present in 88.9% of genomes described (*n* = 1,501) and included 295 unique alleles based on individual single nucleotide polymorphisms and 225 unique mature protein sequences (Fig. 1). Mrp protein sequences ranged from 277 to 430 amino acids in length (Fig. 3) and had an average pairwise identity of 83.2% (Fig. S2B). While Mrp proteins shared functional characteristics with M proteins (22), they were more homogenous in sequence. Unlike the wide distribution of *emm* gene length, genes for *mrp* had more restricted variability of length, since the majority of genes fit into four distinct length classes (Fig. 3).

In place of C-repeat sequences in the C-terminal region of M, Mrp proteins have A-repeat sequences (26, 29) (Fig. S3). These A-repeats were 35 amino acids in length, spanned a region of uninterrupted alpha-helix, and had no intervening sequence. In 95% of Mrp protein sequences, three distinct A-repeats could be identified and in 99% of sequences there were at least two A-repeats. The number of repeats was at least partly responsible for the observed difference in gene lengths. Numbered from the N terminus, the A1 repeats contained 95% pairwise identity, and the A2 and A3 repeats contained 99% pairwise identity. When aligned, all A repeat sequences contained 74% average pairwise identity.

10.1128/mSphere.00806-19.3 FIG S3 Mrp A repeat and Enn C repeat consensus sequences. Sequence logos generated by Geneious based on multiple sequence alignments of the A- and C-repeat regions of Mrp (A) and Enn (B) proteins, respectively. Letter height is determined based on the likelihood of the presence of the amino acid in each position. Sequences are highly homogenous and contain the leucine residues essential for correct coil formation. Download FIG S3, PDF file, 0.3 MB. Copyright © 2020 Frost et al. 2020 Frost et al. This content is distributed under the terms of the Creative Commons Attribution 4.0 International license.

### *enn*.

An *enn* gene was present in 87.1% of genomes (*n* = 1,470), and of these, there were 352 unique alleles. The genes encoded 276 unique mature protein sequences following removal of signal sequences and cleaved regions (Fig. 1). Mature proteins ranged in length from 199 to 346 amino acids (Fig. 3) and had an average pairwise identity of 68.6% (Fig. S2B). Enn proteins therefore presented genetic diversity that is intermediate between the high diversity of M and the low diversity of Mrp and were generally the smaller of this trio of protein families (Fig. 3).

The repeat region of Enn proteins contained C-repeats (30) which are predicted to form alpha-helices disrupted by small regions of random coil and divided by linker regions of 7, 14, or 28 amino acids (Fig. S3). The repeats were less homogenous than the A repeats of Mrp: the C1 repeat was present in 100% of sequences and had 94% sequence identity, the C2 repeat was present in 94% of sequences and had 94% sequence identity, and the C3 repeat was present in 37% of sequences and had 93% sequence identity. The number of repeats present and the combination of linker regions had a large effect on the protein lengths.

Following the variable region at the N terminus, Enn proteins had either an EQ-rich central core (*n* = 154/278; 55% of sequences) with significant similarity to the analogous region in M proteins or, in 39% of sequences (*n* = 109/278), an 18-amino-acid consensus sequence (EKEKEDLKTTLAKTTKEN). There was greater sequence similarity between the N-terminal 50 first amino acids from Enn proteins with the 18-amino-acid core than EQ-rich cores, with 58 and 34% pairwise identities, respectively.

### Sequence similarities across different regions of proteins.

All M and M-like protein sequences were preceded by a signal peptide, typically 41 amino acids in length. The sequences were highly homogenous within each protein family and only slightly less so between the different families (Table 2). The most C-terminal part of the proteins contained the LPXTG sortase motif, which allows attachment of the protein to the bacterial cell wall. This was also the region of the most sequence homogeneity in the mature M and M-like proteins, and all proteins became increasingly heterogeneous more distally (Fig. S2B).

**TABLE 2:** Amino acid sequence identities in different regions of M and M-like proteins^*a*^

Protein(s)	Amino acid identity (%)			
	Signal peptide	First 50 amino acids	51st to repeat region	Repeat region to LPXTG
Mrp	98.0	43.2	91.6	96.1
Emm	81.8	15.0	41.4	87.9
Enn	96.2	27.3	56.2	93.7
Mrp + Emm + Enn	78.4			59.2

a The variability between protein families was too great in the regions from the first amino acids of the mature protein to the repeat regions to perform a meaningful multiple alignment across the three protein families.

### Expression analysis.

**FIG 4:**
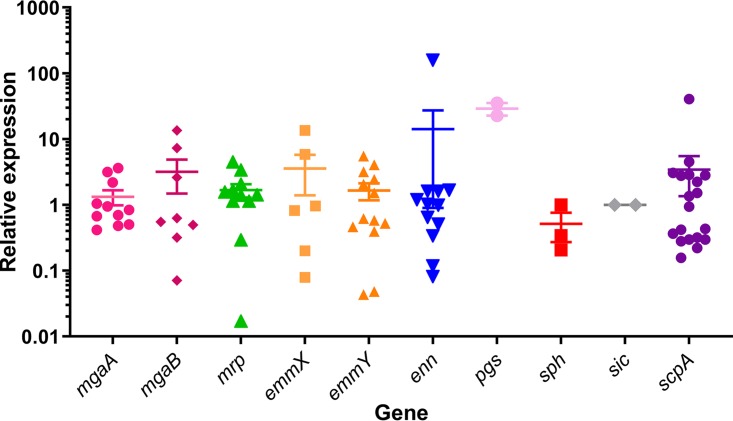
Expression analysis of Mga regulon genes. cDNA from 19 isolates grown to mid-log phase in rich medium were analyzed for the expression of Mga regulon genes. The isolates were selected to be representative of all possible Mga regulon configurations and *emm* cluster diversity where possible. Primers were designed to amplify all members of the gene family where possible (*mrp*, *enn*, *pgs*, *sph*, and *scpA*) and to amplify a subset where sequence diversity necessitates. The dot plot symbols represent the mean value of the four qPCR analyses for each isolate, and the error bars represent the standard errors for all isolates for each gene.

To better characterize expression of the Mga regulon components, gene expression was analyzed during exponential growth of 19 representative isolates grown in rich broth, conditions known to maximize *emm* gene expression (20) (Table S1). The expression of all genes was observed at levels comparable to *emm*, with similar variability (Fig. 4).

10.1128/mSphere.00806-19.5 TABLE S1 Details of isolates used for expression analyses. Isolates used for expression analyses were selected first to be representative of the different Mga regulon configurations identified in this study and of different *emm* clusters. All protein-coding genes present in the different Mga regulon configurations were examined for the ability to be expressed, in more than one isolate when possible. The isolates included in the large genomic study by Davies et al. are indicated by asterisk (*). Download Table S1, DOCX file, 0.01 MB. Copyright © 2020 Frost et al. 2020 Frost et al. This content is distributed under the terms of the Creative Commons Attribution 4.0 International license.

### *In silico emm-*typing ambiguities.

Using the current CDC *emm*-typing database, there were 2,192 *emm*-typing sequences present in the analyzed Mga regulons. After analyses of positional information and the presence of gene-defining oligonucleotide probes (Table 1), as well as BLAST searches, it was determined that 529 of the *emm*-typing sequences were present in genes other than *emm* . The presence of two *emm*-typing sequences in two genes in the same locus (the *emm* gene and a non-*emm* gene) has high potential for mistyping. *emm* sequencing regions for *emm* types *emm*134.1 and *emm*226.0 were present in 20 *sph* genes encoding three distinct protein H alleles. Sequencing regions for the *emm* types *emm*141.0 and *emm*156.0 were also present in 44 *mrp* genes encoding nine distinct Mrp proteins. The remaining 465 *emm* sequence typing regions in non-*emm* genes were in *enn* genes (Table S6). Utilizing whole-genome-sequencing (WGS) data to derive *emm* types of GAS is becoming increasingly common practice; however, there is the possibility for the detection of an *emm*-like gene in place of the *emm* gene (9). A well-curated database of *emm*-typing sequences is essential to reduce potential mistyping using WGS and bioinformatic pipelines. This study utilized a globally informed whole-genome-based platform to thoroughly refine the *emm* sequence database to differentiate PCR-derived *emm* types into those relating to *mrp*, *enn*, and *sph* genes. Of note, 18 different *emm*-typing sequences were not found in *emm* genes but only in *mrp*, *enn*, or *sph* genes. Athey et al. previously identified 12 of these *emm* types (9), and in this work we identified *emm*141 in *mrp*63, *emm*134.1, and *emm*226 in three *sph* genes and *emm*203, *emm*134, and *emm*166 in eight, seven, and four *enn* genes, respectively. Such sequences have been removed from the *emm* sequence typing database and correctly renamed in the appropriate M-like database on the website of the GAS reference laboratory at the CDC (https://www2a.cdc.gov/ncidod/biotech/strepblast.asp).

### Chimeric proteins.

Chimeric *emm* genes containing the N-terminal portion of the *emm* gene and C-terminal portion of the *enn* gene were present in 17 genomes belonging to six different *emm* types (*emm* types 4 [*n* = 12], 9, 44, 58, 73, and 82). These genes contained the 5′ *emm* probe and the *enn* probe at their 3′ ends (Fig. S4). This phenomenon was recently described in M4 isolates (31). Of note, all belonged to a specific Mga configuration with *mrp* and *emm* genes and were exceptions in their *emm* type.

10.1128/mSphere.00806-19.4 FIG S4 Production of chimeric M/Enn proteins. Schematic of possible mechanisms of action resulting in the chimeric *emm/enn* genes found in 13 genomes. Recombination Detection Program (RDP) v4.97 was used to identify potential parent and recombinant sequences using a collection, including predicted chimeric genes and the *emm* and *enn* genes from the same *emm* types that contained both genes separately and the five novel chimeric genes were detected as well as likely *enn* parent genes (A). Software was able to predict the parent alleles of the chimeric gene, in some instances with high statistical probability (B) and identify the breakpoint in the alignment based on pairwise identity between the sequences (C). Triangles indicate the location of gene-specific probes, notably the absence of the 3′ *emm* (yellow) probe in the recombinant gene but presence of both the 5′ *emm* (red) and *enn* probes (dark blue). Download FIG S4, PDF file, 0.05 MB. Copyright © 2020 Frost et al. 2020 Frost et al. This content is distributed under the terms of the Creative Commons Attribution 4.0 International license.

## DISCUSSION

This study provides for the first time a comprehensive, genome-based, genetic description of the GAS Mga regulon and, in particular, the M-like proteins Mrp and Enn. We also provide a molecular definition using conserved oligonucleotide probes for *emm* and *emm*-like genes that allows for proper identification and will improve strain typing.

With the increasing adoption by public health laboratories of WGS for GAS typing in place of PCR sequence typing of the *emm* gene, it is critical to differentiate between *emm* and *emm-*like genes. Although the current system utilizing the 5′ *emm* probe is efficient for detection of *emm* genes (7), this could be further improved by incorporating the 3′ *emm* probe and the other gene family specific probes described in this study into a pipeline for *emm* gene typing. Updating the *emm*-typing collection to reflect the *emm* or *emm-*like genes further decreases the risk of identifying the incorrect gene or detecting two “*emm*” genes in a single genome.

Approximately 85% of genetically diverse GAS genomes were found to encode M, Mrp, and Enn proteins; this is striking because, compared to M, Mrp and Enn have been relatively poorly characterized to date. The retention of these genes suggests an important survival advantage, since pathogenic bacteria are under strong selection pressures. Isolates encoding Mrp and Enn are epidemiologically relevant, causing more than half of global GAS infections (Fig. 2), particularly in developing nations and in the indigenous populations of Australia and New Zealand. Indeed, in the latter populations D4 strains, which all encode the trio of M, Mrp, and Enn proteins, are considered endemic and have been linked to the development of rheumatic fever following skin infection (32, 33). M proteins from D4 strains are relatively small and have been shown to not induce a high M-type-specific antibody response (34). In these cases, it is conceivable that the M-like proteins perform roles otherwise performed by M proteins. The *emm* type is predictive of the composition of the Mga in many instances. This suggests the regulon has evolved as a whole in order to fill a functional niche. No D4 cluster M protein has been shown to bind fibrinogen (35); however, all Mrp proteins have one or two fibrinogen-binding motifs (36), and all D4 *emm*-types contain an *mrp* gene. In high-income settings where specific *emm* types, such as M1 and M12, are responsible for the majority of infections, the proportion of M-like protein-producing strains would be lower (8, 37).

In contrast to previous studies which found very low expression of *mrp* and *enn* genes compared to *emm* genes (20), we observed, under the growth conditions described here, high expression of all *emm* and *emm*-like genes in at least one isolate. Importantly, all genes present in core Mga regulons were capable of being transcribed.

The emergence of a range of *emm* types deriving from possible gene fusion between *emm* and *enn* genes suggests this may be a significant mechanism for the bacteria to alter function or evade immune recognition. This phenomenon, recently identified in M4 isolates (31), appears to have occurred in diverse *emm* types in the United States, a high-income-nation setting where there is low diversity of circulating *emm* types (38, 39).

The prevalence of *emm*-like genes, in addition to their genetic similarities and comparable expression with *emm* genes, indicates the importance of their encoded proteins to GAS virulence. The results presented here will thus aid further genetic and biological characterization of the Mga regulon in order to better understand its role in virulence and vaccine development.

## MATERIALS AND METHODS

### Genome collection and global epidemiological data.

We analyzed Mga regulon genes in a 2,083-GAS genome database representative of worldwide geographic and clinical diversity (25). A previously published global database of GAS infection (38) was used to assess the frequencies of each Mga configuration among global infections.

### Bioinformatics.

The Mga regulon was derived from 2,083 GAS genome assemblies (25) based on the coordinates of the *mga* and *scpA* genes. Mga regulons that were not contained within a single contig (395/2,083) were excluded from the analysis. Annotation was performed using Geneious 10.1.2 based on gene orientation and BLAST searches to initially define *emm*, *enn*, and *mrp* gene families. To facilitate gene identification and solve nomenclature ambiguities, nucleotide probes to identify *emm*, *enn*, and *mrp* were developed based on regions of high sequence identity, with high sensitivity and specificity for each gene family. We also used the previously described *emm*-typing sequence as an *emm* probe (7). The specificity of probes was determined by Geneious 10.1.12 motif search within whole GAS genomes. Alleles with sequence ambiguities and frameshift mutations resulting in truncations were excluded from unique gene and protein sequence analyses (Fig. 1). Unique genes were differentiated by single nucleotide variations, and genes that produced proteins with the same amino acid sequences were annotated as subtypes (e.g., mga13.0 and mga13.1). The *emm*-typing database available from the CDC website (www.cdc.gov/streplab) was used to annotate *emm* genes and to search for *emm*-typing sequences within *emm*-like genes.

Mature protein sequences of M and M-like proteins were derived by removing the 41 to 42 amino acid signal peptide based on the EMBOSS 6.5.7 tool sigcleave at the amino terminus and the cleaved region following the threonine of the sortase LPXTG signal at the carboxy terminus. Repeat regions were identified by comparison to published sequences for M and M-like proteins (3, 30). Domains were defined as the N-terminal 50 amino acids, the 51st amino acid to the beginning of A- or C-repeat sequences and the repeat sequences to the mature protein’s last residue (22).

Multiple alignments were performed with the MAFFT program using the global pairwise iterative refinement (G-INS-i) method which uses the Needleman-Wunsch algorithm and BLOSUM62 scoring matrix (40). The percent identities at each position along amino acid sequences were graphed using Geneious 10.1.2, and genetic distances between groups of *mga* alleles were calculated using MEGA version X (41). RDP v4.97 (42) was used to detect recombination events and map gene breakpoints.

### Expression analysis.

A representative collection of 19 GAS strains were selected for expression analyses, to include a diverse array of Mga regulon configurations and *emm* clusters (Table S1). Reverse transcriptase quantitative PCR was performed on the 19 representative GAS strains. Bacteria were grown at 37°C with 5% CO_2_ in Todd-Hewitt broth (Carl Roth, Karlsruhe, Germany) with 0.5% yeast extract (Carl Roth) until exponential phase (optical density at 600 nm, 0.4 to 0.6), and 1 ml of culture was harvested at 5,000 × *g* for RNA extraction. Bacteria were washed once with distilled water, and pellets were lysed with enzymatic lysis buffer consisting of 9.5 mg/ml lysozyme, 20 mM Tris-HCl, 3 mM EDTA, and 1% Triton X-100. Further lysis and separation of aqueous phase was achieved using PureZOL RNA isolation reagent (Bio-Rad; 732-6880), and RNA extraction was performed according to manufacturer’s instructions (Aurum Bio-Rad; 732-6870). After extraction, genomic DNA contamination was removed using Turbo DNase treatment (Invitrogen; AM1907), and the RNA yield and purity was estimated using a QuickDrop spectrophotometer (Molecular Devices). Reverse transcriptase (NEB; E6560) was performed using approximately 300 ng of RNA and a 6 μM concentration of the random primer mix provided for 1 h at 42°C after an initial incubation for 5 min at 25°C. Target genes from 5 μl of cDNA were amplified in 20-μl reactions with Luna Universal SYBR qPCR master mix (NEB; M3003) in a Bio-Rad CX96 real-time PCR detection system with conditions as follows: 95°C for 60 s, followed by 40 cycles of 95°C for 15 s and 60°C for 30 s. Dissociation curves were calculated for each reaction to confirm product specificity. No-reverse-transcriptase and no-template controls were performed for each extraction and each pair of primers. Results were analyzed with qBase+ (Biogazelle) software, and the relative expression compared to *recA* was calculated for each sample. Specific primers were used (see Table S2), and amplification efficiencies were calculated. cDNA was produced twice from each strain, each sample was analyzed twice by qPCR, and the four relative expression values were averaged.

10.1128/mSphere.00806-19.6 TABLE S2 Primers used in expression analyses by RT-qPCR. Primer efficiencies were calculated using known concentration of genomic DNA of a subset of strains for each gene (shown in boldface). Based on the composition of the Mga regulon of the strain, expression was examined using universal primer sets where possible. The *mga* gene from strains that encoded the trio of Mrp, M, and Enn proteins was amplified by *mgaB* primers, and the *mga* gene from the other strains was amplified by *mgaA* primers. Three pairs of primers were used for the different *emm* genes: *emmX* for strains from clade X *emm* clusters (10), *emmY-1* for A to C *emm* pattern strains from clade Y *emm* clusters, and *emmY-2* for clade Y *emm* clusters that encoded *emm*-like genes, plus the M6 isolate. Download Table S2, DOCX file, 0.01 MB. Copyright © 2020 Frost et al. 2020 Frost et al. This content is distributed under the terms of the Creative Commons Attribution 4.0 International license.

10.1128/mSphere.00806-19.7 TABLE S3 Proteins and alleles present in the Mga regulons of all genomes analyzed in this study. Download Table S3, XLSX file, 0.2 MB. Copyright © 2020 Frost et al. 2020 Frost et al. This content is distributed under the terms of the Creative Commons Attribution 4.0 International license.

10.1128/mSphere.00806-19.8 TABLE S4 *mrp* and *enn* alleles in this collection. Download Table S4, XLSX file, 0.3 MB. Copyright © 2020 Frost et al. 2020 Frost et al. This content is distributed under the terms of the Creative Commons Attribution 4.0 International license.

10.1128/mSphere.00806-19.9 TABLE S5 Distribution of *emm* patterns, clusters, and types in different Mga regulon configurations. The vast majority of genomes in this collection encoded the trio of M and M-like proteins and included *emm* types from many multiprotein clusters, some single protein clusters from both clades X and Y and four *emm* types that have yet to be assigned a cluster (N/C). The remaining configurations were more restricted to both *emm* type and cluster. Strikingly, all genomes that encoded *pgs* belonged to the E3 cluster, and all genomes that did not encode *mrp* or *enn* belonged to only 15 different *emm* types. Global infection data are from Steer et al. (LID, 2009). Download Table S5, XLSX file, 0.02 MB. Copyright © 2020 Frost et al. 2020 Frost et al. This content is distributed under the terms of the Creative Commons Attribution 4.0 International license.

10.1128/mSphere.00806-19.10 TABLE S6 M-like genes with *emm*-sequence typing. *emm* sequence typing regions were detected in many *emm*-like genes, with 100% sequence identity, predominantly in *enn* genes. The 19 *emm* types (plus nine subtypes) largely belong to either the E5 *emm* cluster or were not assigned to an *emm* cluster and aside from emm137.0 were not otherwise present in *emm* genes. Download Table S6, DOCX file, 0.01 MB. Copyright © 2020 Frost et al. 2020 Frost et al. This content is distributed under the terms of the Creative Commons Attribution 4.0 International license.
