# Supplementary figures and images for: Analysis of Global Collection of Group A Streptococcus Genomes Reveals that the Majority Encode a Trio of M and M-Like Proteins
Source: mSphere. 2020 Jan 8;5(1):e00806-19. doi: 10.1128/mSphere.00806-19 (PMC6952200; doi:10.1128/mSphere.00806-19)

Lengths of Mga regulon genes

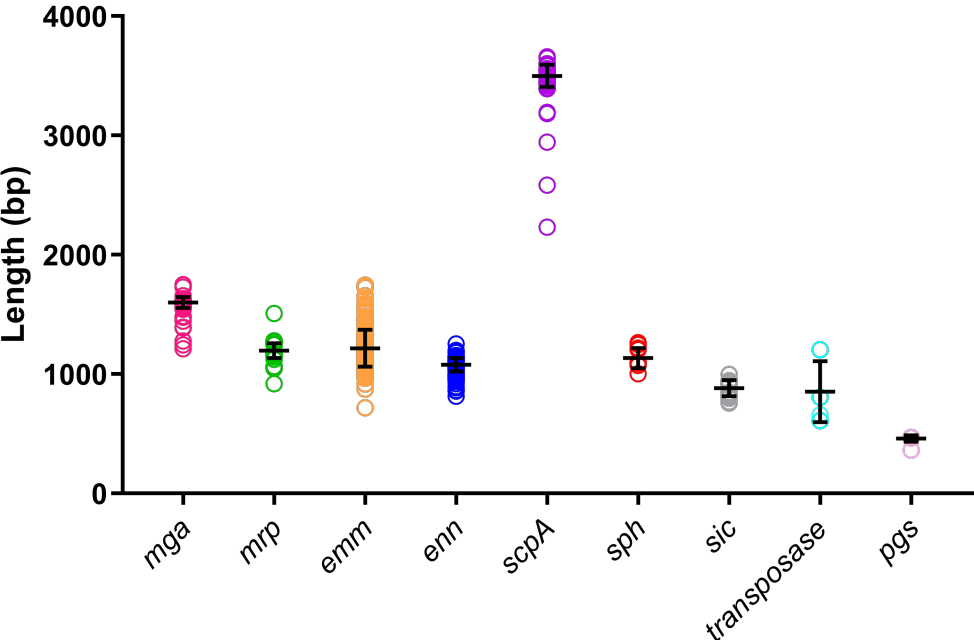

Supplement: FIG S1 [file mSphere.00806-19-sf001.pdf]

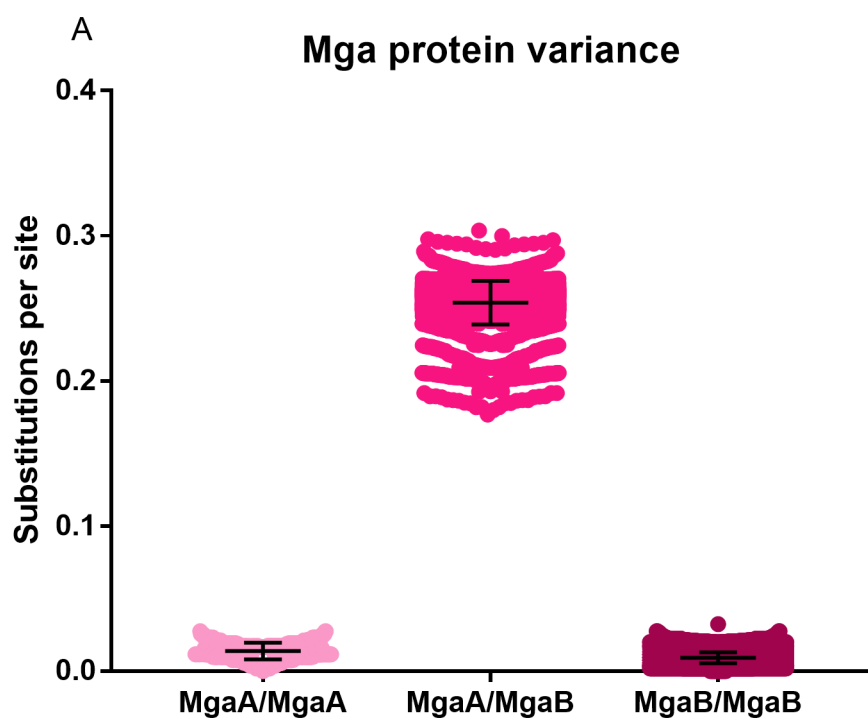

B

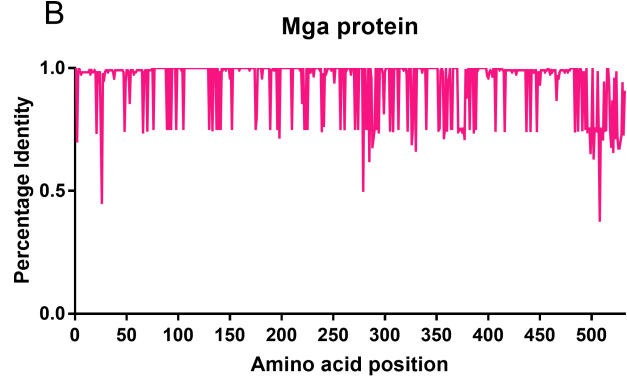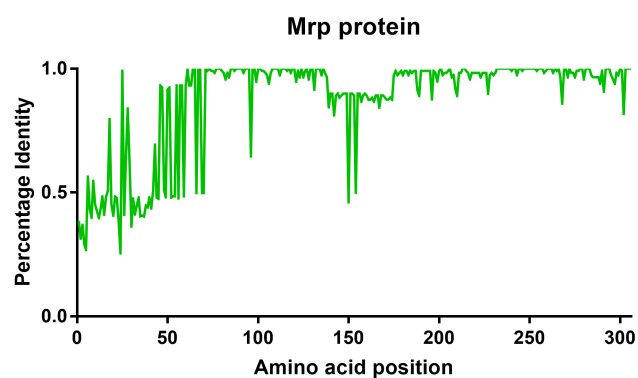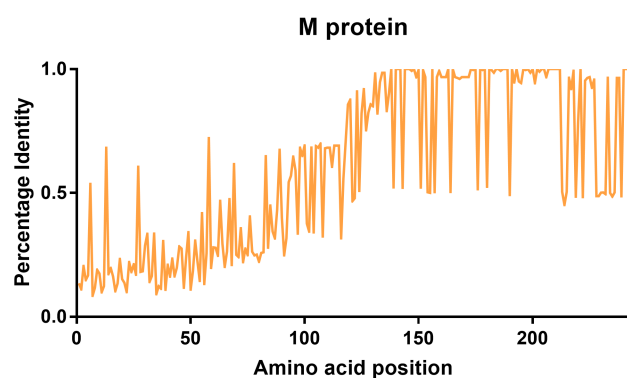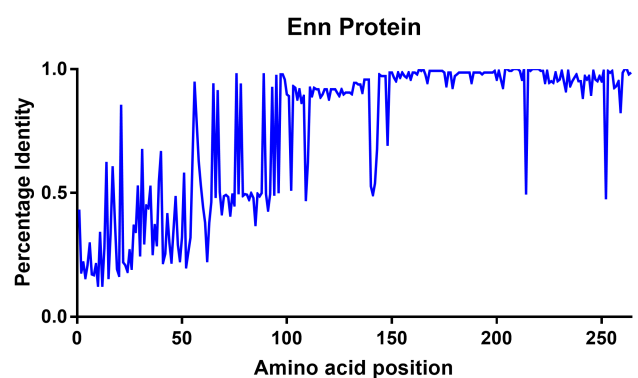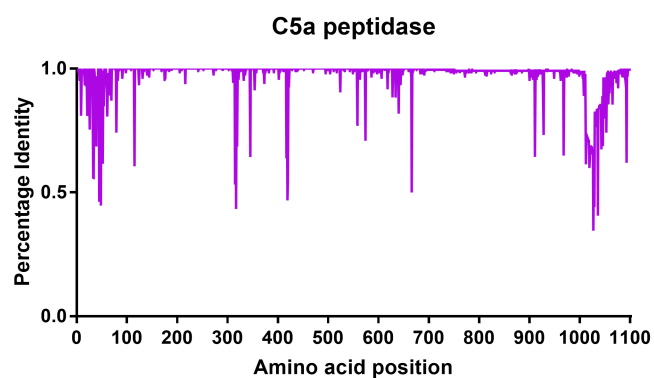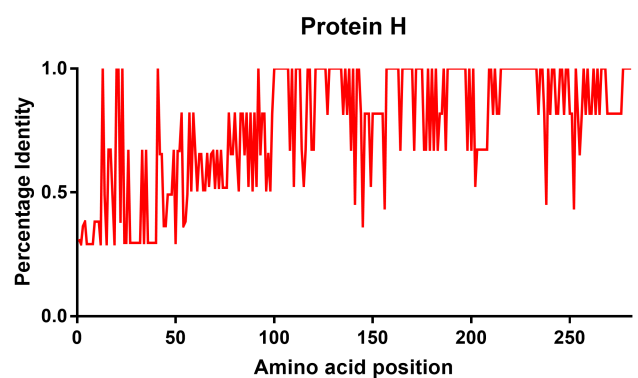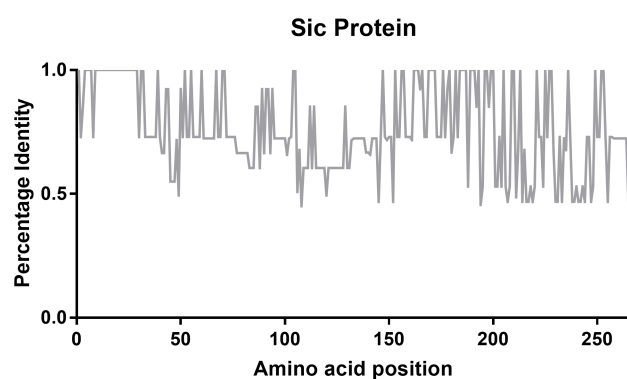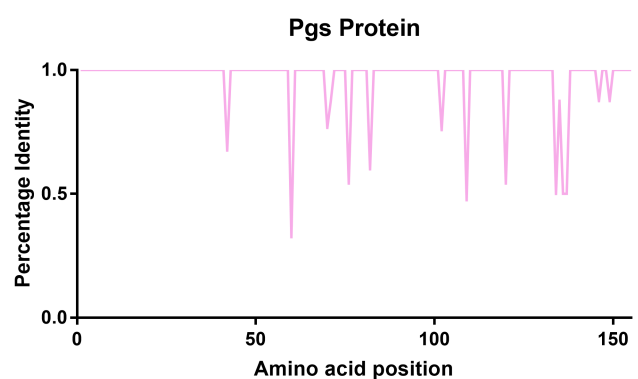

Supplement: FIG S2 [file mSphere.00806-19-sf002.pdf]

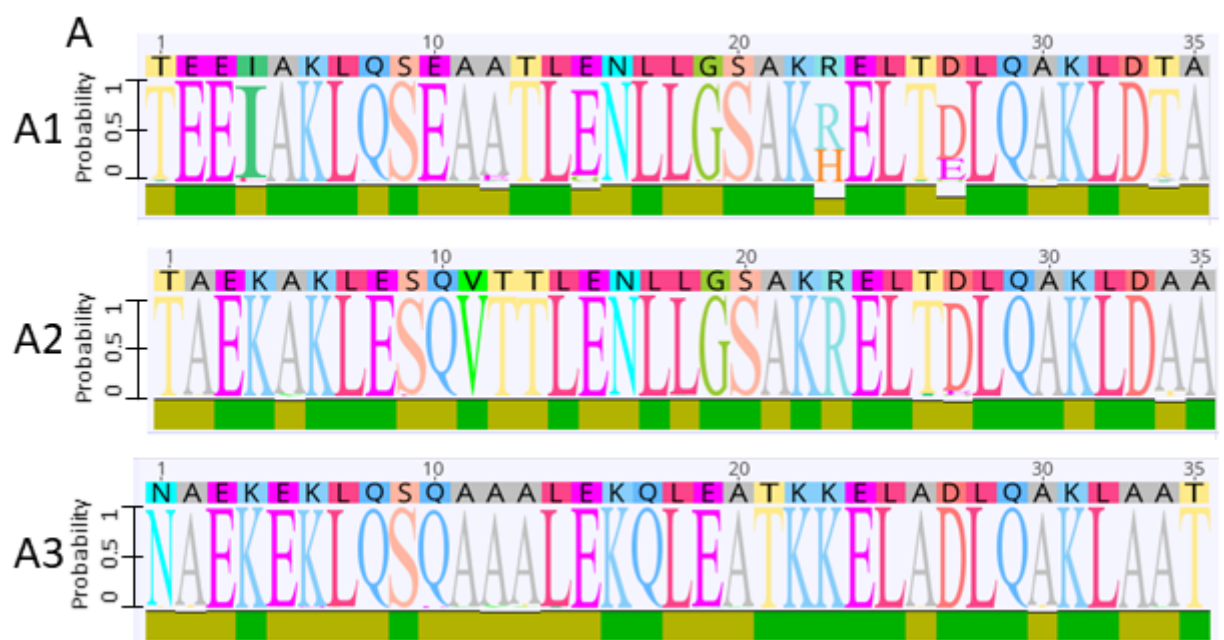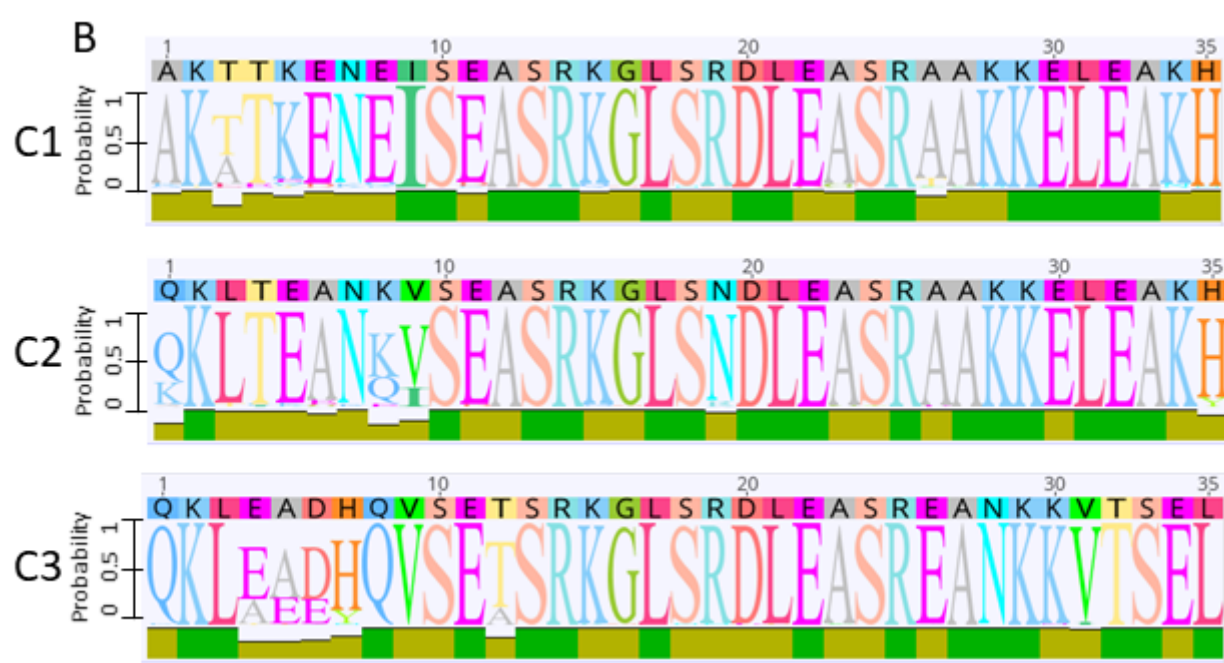

Supplement: FIG S3 [file mSphere.00806-19-sf003.pdf]

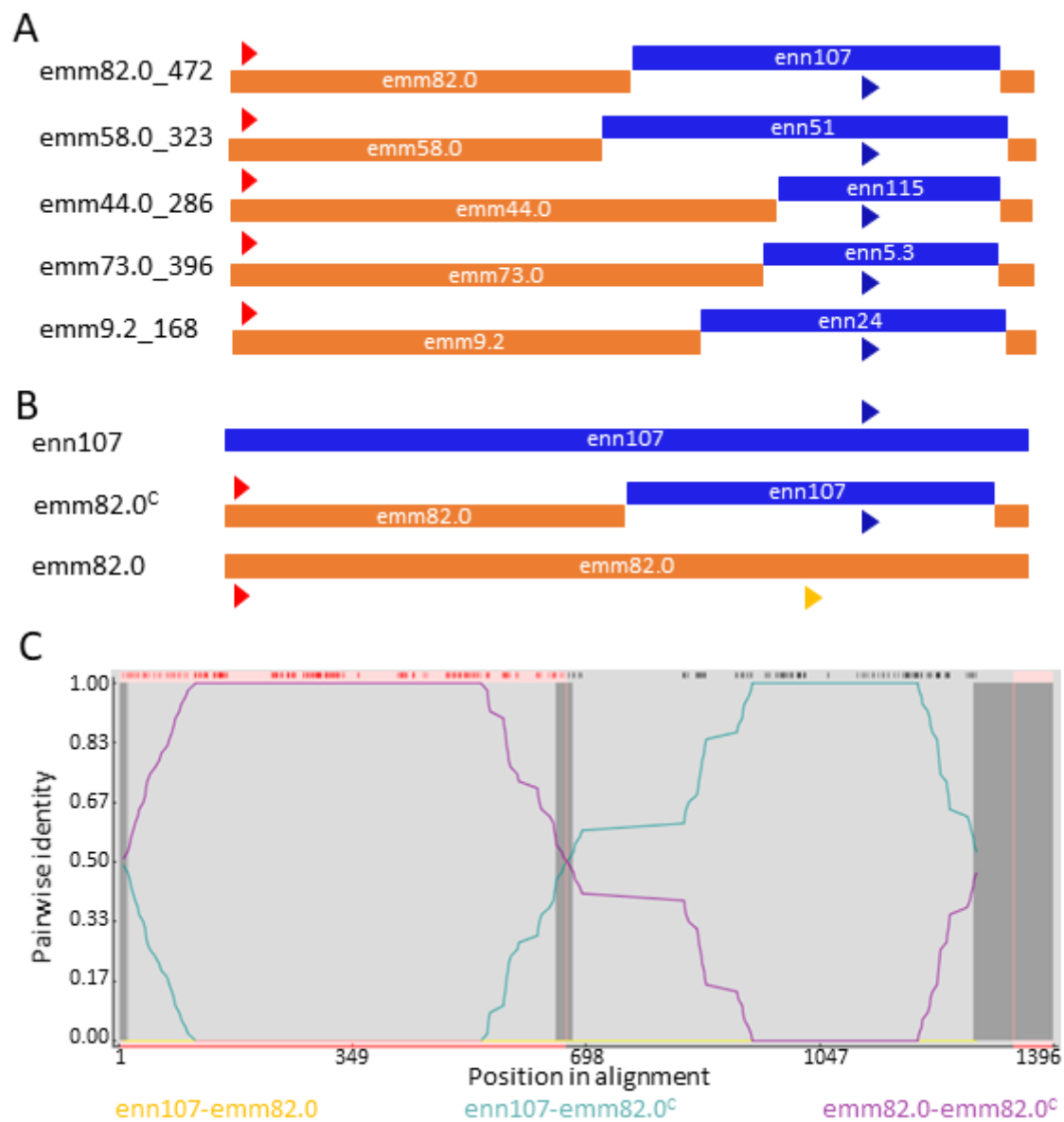

Supplement: FIG S4 [file mSphere.00806-19-sf004.pdf]
