# Supplementary material for: Analysis of Global Collection of Group A Streptococcus Genomes Reveals that the Majority Encode a Trio of M and M-Like Proteins
Source: mSphere. 2020 Jan 8;5(1):e00806-19. doi: 10.1128/mSphere.00806-19 (PMC6952200; doi:10.1128/mSphere.00806-19)
Supplement: TABLE S1 [file mSphere.00806-19-st001.docx]

| **Strain ID** | **M-type** | **emm cluster** | **Mga configuration** | **Country of origin** | |
| --- | --- | --- | --- | --- | --- |
| 31126V4S1 | M222 | Clade Y outlier | mga-emm-enn-scpA | Fiji | [43] |
| 4152 | M3 | A-C5 | mga-emm-scpA | Belgium | [42] |
| 4166 | M6 | Single protein cluster Clade Y | mga-emm-scpA | Belgium | [42] |
| 4048 | M12 | A-C4 | mga-emm-sic-scpA | Belgium | [42] |
| 4235 | M1 | A-C3 | mga-emm-sic-scpA | Belgium | [42] |
| 31027V4S1* | M57 | Single protein cluster Clade Y | mga-emm-sph-scpA | Fiji | [25, 43] |
| I66/Bel006* | M19.4 | Single protein cluster Clade Y | mga-emm-sph-scpA | Brazil | [25, 42] |
| 33141V4T1 | M238 | A-C3 | mga-emm-sph-sic-scpA | Fiji | [43] |
| 4460 | M102 | E4 | mga-mrp-emm-enn-scpA | Belgium | [42] |
| I36 | M74.0 | Single protein cluster Clade Y | mga-mrp-emm-enn-scpA | Brazil | [42] |
| I85 | M83.1 | D4 | mga-mrp-emm-enn-scpA | Brazil | [42] |
| NS13* | M53.0 | D4 | mga-mrp-emm-enn-scpA | Australia | [25] |
| NS178* | M54.1 | D1 | mga-mrp-emm-enn-scpA | Australia | [25] |
| NS192* | M106 | E2 | mga-mrp-emm-enn-scpA | Australia | [25] |
| NS730* | M90.5 | E2 | mga-mrp-emm-enn-scpA | Australia | [25] |
| NS80* | M70.0 | D4 | mga-mrp-emm-enn-scpA | Australia | [25] |
| NS88.2* | M98.1 | D4 | mga-mrp-emm-enn-scpA | Australia | [25] |
| I29 | M82 | E3 | mga-mrp-emm-pgs-enn-scpA | Brazil | [42] |
| I75 | M58 | E3 | mga-mrp-emm-pgs-enn-scpA | Brazil | [42] |
