# Supplementary material for: Analysis of Global Collection of Group A Streptococcus Genomes Reveals that the Majority Encode a Trio of M and M-Like Proteins
Source: mSphere. 2020 Jan 8;5(1):e00806-19. doi: 10.1128/mSphere.00806-19 (PMC6952200; doi:10.1128/mSphere.00806-19)
Supplement: TABLE S2 [file mSphere.00806-19-st002.docx]

| Gene | Sequence (5’-3’) | Efficiency % | emm-type of strains utilised |
| --- | --- | --- | --- |
| *mgaA* | F-AGCTCAATCTCAGCATCACCAA  R-TATGGAGGGTTACGGCAACTTC | 95.72 | 1**, 3, 6,** 12**,** 19.4**, 57, 222, 238** |
| *mgaB* | F-CAACGGGCTGTCGAAAAGTG  R-GTGACGCTGAGATTGAGGCT | 94.93 | 53**, 54, 58, 70, 74, 82,** 83**, 90, 98, 102,** 106 |
| *mrp* | F-TGACTGACTTGCAAGCTAAGCT  R-TTCACGCTTAGCTGAACCTAGG | 93.65 | **53, 54, 58,** 70**, 74, 82, 83,** 90**,** 98**, 102,** 106 |
| *emmX* | F-GCAAATAGCAAACTTCAAGCCC  R-CTTGCTTGTAACTCAGCTTTTTCTT | 95.58 | 58**, 70, 82, 90, 102,** 106 |
| *emmY*  *1* | F-TCACAAACCCCTGATGCAAAAC  R-TGGTGCTTTGTTTTGGTTAGG | 106.8 | 1**, 3, 12,** 19.4**, 57, 222, 238** |
| *emmY*  *2* | F-TCAGAAGCAAGCCGTAAAG  R-TCAAGTTCAGCAGTCAAGTTTG | 105.5 | **6,** 53**,** 54**,** 74**, 83, 98** |
| *enn* | F-TCAACTCTCAGCGCTTGAAGAA  R-AGGGCTTTTCCTTGTGCATCTA | 97.79 | **53, 54, 58,** 70**, 74, 82, 83, 90, 98, 102, 106, 222** |
| *sph* | F-GGCTGATCACCAAGCCCTAG  R-AGCGCTGAGAGTTGAGCTTT | 92.66 | 19.4**, 57, 238** |
| *sic1* | F-TCCCAAGTGAACCTCGTGTG  R-AAGCCAGCTGAAAACCCTCT | 93.91 | 1 |
| *sic12* | F-TCTGGTGAGCCTATTCCGGA  R-ACTGCTGTTCCCCATTCCTT | 94.79 | 12 |
| *sic238* | F-TGGATTATCTGGCCCCTCTGA  R-GGAGTCTCTGGAGCTTGTGG | 82.80 | **238** |
| *pgs* | F-AGCCATCAAGTTTGTGGGCT  R-TGGAGTAGGTCTAGGCCCAC | 95.54 | 58**, 82** |
| *scpA* | F-CCCGGCCAAGATATTTTGTCATC  R-AGACGCTCTGATGGTGTCATATC | 93.82 | **1, 3, 6, 12, 19.4, 53,** 54**, 57,** 58**, 70, 74, 82, 83,** 90**, 98, 102,** 106**, 222, 238** |
| *recA* | F-CGACTGTGGCTTTACATGCTGTA  R-TGCTCGGCATCGATAAAGG | 99.23 | **1, 3, 6, 12, 19.4, 53, 54, 57, 58, 70, 74, 82, 83, 90,** 98**, 102, 106, 222, 238** |
