# Supplementary material for: Analysis of Global Collection of Group A Streptococcus Genomes Reveals that the Majority Encode a Trio of M and M-Like Proteins
Source: mSphere. 2020 Jan 8;5(1):e00806-19. doi: 10.1128/mSphere.00806-19 (PMC6952200; doi:10.1128/mSphere.00806-19)
Supplement: TABLE S6 [file mSphere.00806-19-st006.docx]

| **M-like allele** | **emm-type** | **emm-cluster** | **M-like allele** | **emm-type** | **emm-cluster** |
| --- | --- | --- | --- | --- | --- |
| enn266 | emm166.1 | E2 | enn47 | emm236.2 | M236 |
| enn267 | emm166.1 | E2 | enn1 | emm236.3 | M236 |
| enn260 | emm166.2 | E2 | enn2 | emm236.3 | M236 |
| enn261 | emm166.2 | E2 | enn216.1 | emm138.0 | N/A |
| enn255 | emm134.0 | E5 | enn218 | emm138.0 | N/A |
| protein H1 | emm134.1 | E5 | enn174 | emm138.1 | N/A |
| enn252.0 | emm134.2 | E5 | mrp63 | emm141.0 | N/A |
| enn252.1 | emm134.2 | E5 | enn115 | emm149.2 | N/A |
| enn254 | emm134.2 | E5 | enn125 | emm149.2 | N/A |
| enn256 | emm134.2 | E5 | enn126 | emm149.2 | N/A |
| enn258 | emm134.2 | E5 | enn127.0 | emm149.2 | N/A |
| enn259 | emm134.2 | E5 | enn127.1 | emm149.2 | N/A |
| enn282 | emm137.0 | E5 | enn72 | emm159.0 | N/A |
| enn283 | emm137.0 | E5 | enn73 | emm159.0 | N/A |
| enn334 | emm170.0 | E5 | enn75 | emm159.0 | N/A |
| enn335 | emm170.0 | E5 | enn79 | emm159.0 | N/A |
| enn336 | emm170.0 | E5 | enn80 | emm159.0 | N/A |
| enn337.1 | emm170.0 | E5 | enn81 | emm159.0 | N/A |
| enn341 | emm170.0 | E5 | enn82 | emm159.0 | N/A |
| enn349 | emm170.0 | E5 | enn83 | emm159.0 | N/A |
| enn342 | emm170.1 | E5 | enn288 | emm167.0 | N/A |
| enn248 | emm174.0 | E5 | enn290 | emm167.0 | N/A |
| enn249 | emm174.0 | E5 | enn343 | emm202.1 | N/A |
| enn250 | emm174.0 | E5 | enn344.0 | emm202.1 | N/A |
| enn251 | emm174.0 | E5 | enn344.1 | emm202.1 | N/A |
| enn230 | emm174.1 | E5 | enn345 | emm202.1 | N/A |
| enn234 | emm174.1 | E5 | enn24.0 | emm203.4 | N/A |
| enn235 | emm174.1 | E5 | enn24.1 | emm203.4 | N/A |
| enn236 | emm174.1 | E5 | enn26.0 | emm203.4 | N/A |
| enn240 | emm174.1 | E5 | enn29 | emm203.4 | N/A |
| enn241 | emm174.1 | E5 | enn30 | emm203.4 | N/A |
| enn299 | emm205.0 | E5 | enn31 | emm203.4 | N/A |
| enn300.0 | emm205.0 | E5 | enn32 | emm203.4 | N/A |
| enn300.1 | emm205.0 | E5 | enn33 | emm203.4 | N/A |
| enn303 | emm205.0 | E5 | enn268 | emm240.1 | N/A |
| enn355 | emm205.0 | E5 | enn270 | emm240.1 | N/A |
| enn310 | emm205.1 | E5 | enn277 | emm240.2 | N/A |
| enn311 | emm205.1 | E5 | mrp179.0 | emm156.0 | N/A |
| enn312 | emm205.1 | E5 | mrp179.1 | emm156.0 | N/A |
| enn318 | emm164.3 | M164 | mrp179.2 | emm156.0 | N/A |
| enn319 | emm164.3 | M164 | mrp182 | emm156.0 | N/A |
| enn320 | emm164.3 | M164 | mrp183.0 | emm156.0 | N/A |
| enn323 | emm164.3 | M164 | mrp183.1 | emm156.0 | N/A |
| enn38 | emm236.2 | M236 | mrp183.2 | emm156.0 | N/A |
| enn39 | emm236.2 | M236 | mrp186 | emm156.0 | N/A |
| enn40.0 | emm236.2 | M236 | protein H2 | emm226.0 | N/A |
| enn46 | emm236.2 | M236 | protein H5 | emm226.0 | N/A |
